# Cardiovascular Risk in Rheumatoid Arthritis: Considerations on Assessment and Management

**DOI:** 10.31138/mjr.310824.cri

**Published:** 2024-09-30

**Authors:** Panagiota Anyfanti, Alexandra Ainatzoglou, Elena Angeloudi, Olga Michailou, Kleopatra Defteraiou, Eleni Bekiari, George D. Kitas, Theodoros Dimitroulas

**Affiliations:** 1 Second Medical Department, Hippokration Hospital, Aristotle University of Thessaloniki, Thessaloniki, Greece; 2 Fourth Department of Internal Medicine, Hippokration Hospital, Aristotle University of Thessaloniki, Thessaloniki, Greece; 3 Medical School, Aristotle University of Thessaloniki, Thessaloniki, Greece; 4 Department of Rheumatology, Russells Hall Hospital, Dudley Group NHS Foundation Trust, Dudley, UK; School of Sport, Exercise and Rehabilitation Sciences, University of Birmingham, Birmingham, United Kingdom

**Keywords:** rheumatoid arthritis, cardiovascular risk, cardiovascular risk factors, inflammation

## Abstract

In the context of holistic therapeutic practices, the cardiovascular risk of patients with rheumatoid arthritis (RA) needs to be addressed as a major factor of compromised disease prognosis and increased mortality. The elevated prevalence of cardiovascular disease (CVD) by more than twofold in RA has been attributed, inter alia, to chronic inflammation exacerbating arterial stiffness, increased onset of hypertension, dyslipidaemia and diabetes mellitus, sedentary lifestyle, and antirheumatic drug complications. CVD risk in RA can be currently assessed by practitioners through accessible adapted calculators, but it remains problematic as their diagnostic accuracy is not superior to calculators designed for the general population. Implementation of guideline-oriented personalised interventions remains the cornerstone for cardiovascular risk management in RA. Remarkably, there is lack of a consortium that brings together different health care providers engaged in the care of patients with RA (e.g., rheumatologists, cardiologists, general practitioners, etc), to guide cardiovascular risk assessment and management. This narrative review aims at providing an overview of current CVD risk assessment and management options, highlighting their pivotal role in the comprehensive treatment of RA patients.

## INTRODUCTION

Rheumatoid arthritis (RA) is a systemic chronic inflammatory disease and an autoimmune disorder that primarily affects the joints but may also be accompanied by systemic organ involvement.^1^ A vast amount of evidence strongly suggests that cardiovascular disease (CVD) is to be regarded as a common extra-articular manifestation of RA. As evidenced by large-scale epidemiological studies, the risk of myocardial infarction in RA equals that of diabetes mellitus (DM)^2^ and the overall CVD risk is 50-70% higher compared to the general population, leading to cardiovascular events that substantially compromise RA prognosis and increase mortality.^3^ Following the development of modern treatments that have significantly expanded the therapeutic armamentarium for RA, patients do not die from the disease per se any longer, but are subject to CVD manifestations in the course of the disease that affect life expectancy and limit prognosis. These findings indicate that the screening, prevention and management of CVD is crucial for disease survival and should therefore constitute a major pillar of RA treatment, rather than a secondary objective.^4^

Chronic, subclinical inflammation in RA represents a primary mechanistic model for the development and progression of CVD, as it precedes and accelerates large artery dysfunction, systemic atherosclerosis, and related risk factors.^5^ Hence, a pivotal strategy to abate CVD risk in RA is through disease control. It has been previously established that inflammatory processes activated in RA exacerbate arterial stiffness, affect the lipid profile, and destabilise atheromatic plaques, thus increasing the risk of infarction. Therefore, confining RA inflammation inevitably leads to CVD risk reduction since both disease flare-ups and the accumulating impact of the disease have been correlated with an increased incidence of CVD events.^4^ However, maintaining an exclusively disease-centred approach to CVD risk reduction is prone to neglect other significant and potentially modifiable factors. In particular, prevalence of traditional CVD risk factors is higher among patients with RA compared to the general populations and aggravate their cardiovascular profile. Nevertheless, recent real-world clinical data from the SURF-RA study across three continents confirm that CVD risk factors are common, yet remain underdiagnosed and suboptimally treated among RA individuals.^6^ In this context, a combined approach is warranted that involves strategies towards effective control of comorbidities, along with appropriate management of RA to achieve remission.

Despite the increasing awareness of the problem, several gaps in knowledge remain regarding assessment and monitoring of CVD risk in patients with RA. There is at presence lack of a validated CVD risk calculator to accurately reflect CVD risk in patients with RA, and no specific recommendations exist from relevant scientific societies regarding management of CVD comorbidities in RA. Therefore, in the present narrative review article, we aim to provide an overview of CVD risk assessment and monitoring in RA. Available population-based and disease-specific CVD risk calculators will be summarised, and cardiovascular aspects of RA treatment will be discussed. Special emphasis will be placed upon the significance of traditional modifiable CVD risk factors and their management. To this end, a PubMed search was performed to identify relevant articles using the following medical terms: “rheumatoid arthritis”; “CVD risk”; “hypertension”; “dyslipidaemia”; “diabetes”, and “disease-modifying antirheumatic drugs (DMARDs)”.

## ASSESSMENT AND MONITORING OF CVD RISK IN RA PATIENTS

Considering the excess CVD burden in patients with RA, CVD risk assessment is crucial and should be part of their daily care. Despite major advances in understanding the pathogenesis of increased CVD risk, CVD risk estimation in RA remains problematic. Population-based CVD risk calculation tools tend to underestimate CVD risk, yet neither specifically designed calculators have been proven superior when compared to traditional population-based algorithms.

### The role of traditional population-based risk tools

SCORE is a widely applied CVD risk estimation system, evaluating an individual’s 10-year risk of cardiovascular mortality. It is based on age, gender, smoking habits, total cholesterol levels and systolic blood pressure.^7^ This calculator provides country-specific recalibrations for low, moderate, high and very high-risk populations.^8^ SCORE2 is an updated version of SCORE2 that has been introduced in the 2021 European Guidelines on CVD prevention.^9^ SCORE2 calculates 10-year risk of fatal and nonfatal CV events in apparently healthy individuals aged 40–69 years with risk factors, and a corresponding SCORE2-OP algorithm is also available for older people (age 70–89 years).^10,11^

The original Framingham Heart Study and the Framingham Offspring Study are considered as landmark studies regarding CVD epidemiology and prevention.^12^ Based on data provided from these cohorts, the Framingham Risk Score (FRS) has been developed to estimate the 10-year risk of manifesting clinical CVD (coronary artery disease, Stroke, peripheral vascular disease, congestive heart failure, cardiac death), taking into account traditional CV risk factors.^13^

Moreover, the American College of Cardiology and American Heart Association (ACC/AHA 2013) Pooled Cohort Equation risk calculator is an algorithm that predicts the 10-year risk of ASCVD in patients aged 40–75 years based on traditional risk factors and lipid profile values (TC, HDL-C).^14^ This index did not provide more accurate classification compared to general and disease-specific calculators, as it significantly overestimated the risk of CVD in a cohort of 1796 RA patients without prior CVD during a mean follow-up of 6.9 years.^15^

As these risk tools are designed for the general population, cardiovascular risk is underrated in RA patients by use of the above calculators.^16^ An important point to consider is that population-based risk calculators are focused on men, who are at higher risk of CVD than women, who constitute the major population in RA.^17^ Moreover, risk factors such as high clinical activity, current or chronic inflammatory burden, disease progression and seropositivity are not evaluated.^18^ Thus, RA-adapted risk calculators have been developed as further discussed below.

### CVD risk calculators developed specifically for patients with RA

Efforts have been made to adjust general cardiovascular risk estimators to patients with Rheumatoid Arthritis. EULAR recommended that risk score models should be altered for RA patients after multiplication by the factor of 1,5 if two of the three following criteria are fulfilled: i) disease duration >10 years, ii) RF or ACPA positivity, iii) extra-articular manifestations.^19^ It is further recommended to estimate CV risk at least once every 5 years and reassess after significant changes in antirheumatic treatment.^20^ However, EULAR multiplier successfully reclassified only a few RA patients above treatment threshold for CVD.^15^ The QRISK-2 calculator is considered to be one of the most important tools in estimating CV risk in the United Kingdom.^21^ This algorithm includes RA as a separate cardiovascular risk factor for people between 24 and 84 years old. It takes into consideration several risk elements such as age, gender, ethnicity, smoking, systolic blood pressure, total cholesterol to high-density lipoprotein ratio, family history of CVD, diabetes, atrial fibrillation, chronic kidney disease, etc. In 2017, QRISK2 updated to QRISK3 adding to the equation special risk factors such as migraine, corticosteroids, atypical antipsychotics, HIV infection, erectile dysfunction, etc.^22^

The Reynolds Risk Score (RRS) is an estimator that incorporates the traditional risk factors, family history and high sensitivity C-reactive protein (hs-CRP) levels, which makes it more suitable for patients with inflammatory-mediated immune disorders. However, relevant studies have questioned the credibility of this calculator for patients with RA as it miscalculates CVD risk in RA.^23^

The Expanded Cardiovascular Risk Prediction Score for Rheumatoid Arthritis (ERS-RA) takes into account both traditional and RA-specific risk factors such as clinical activity of the disease (Clinical Disease Activity Index), disability (Health Assessment Questionnaire Disability Index), disease duration and corticosteroid use.^24^ Further research needs to be done to evaluate the efficacy of the calculator in early RA and long disease duration.^25^ Despite the fact that several risk calculators have been designed in order to predict and prevent CVD in RA patients, none of them is precise enough to accurately reflect CVD risk of RA individuals.^16^ RA-adjusted algorithms are not more efficient than traditional risk tools, because they often refer to a certain population and with restricted systematic characteristics. Other possible factors are selection bias, the mixture of risk factors and the structure of the study. Therefore, it is necessary to design novel algorithms specifically addressed to patients with RA. Studies that compare RA-adapted to general population-based calculators might help to reach a conclusion. Nonetheless, imaging techniques such as CMR and carotid ultrasound are a very useful tool, because they are capable of recognising cardiovascular alterations in early stages.^26^

In conclusion, every patient should be approached individually and comprehensively so as to assess and monitor correctly their CVD risk, taking into account classical CVD risk factors as well as disease-related characteristics (e.g., flares, inflammatory burden, disease activity and duration, etc.). There is an ultimate need for doctors from different specialties and countries engaged in the care of RA patients to reach a consensus on CV risk assessment and monitoring.

## MANAGEMENT OF CVD RISK IN RA

**Figure 1.**
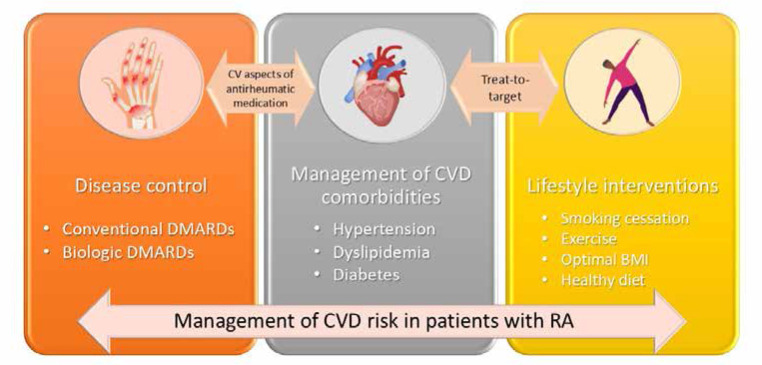
Effective management of cardiovascular disease (CVD) risk in rheumatoid arthritis (RA) requires a multi-directional approach: 1) adequate management of inflammation and disease control with either conventional or biologic disease modifying antirheumatic drugs, or their combination, taking into account their potential cardiovascular effects, 2) early identification and adequate control of cardiovascular comorbidities (e.g., hypertension, diabetes, dyslipidaemia) with conventional medication (antihypertensive, hypoglycaemic and/or hypolipidemic treatment), and 3) lifestyle interventions aiming at smoking cessation, maintaining optimal body mass index, and adopting healthy diet and systematic exercise. It is important to assess CVD risk and screen for CVD comorbidities regularly in patients with RA, especially at times of flares or modification of antirheumatic treatment (upregulation or downtitration).

The emergence of cardiovascular events remains multifactorial, and this is particularly true for autoimmune rheumatic disorders. In this context, effective CVD risk management in RA requires a multi-directional approach, as presented in **Figure 1**. It is highlighted that RA patients need to be stratified according to several parameters in order to be treated appropriately. The risk-enhancing effects of disease duration and activity need to be considered further, as traditional risk factors alone are not adequate to explain the high CV risk in terms of differentiating them from the non-autoimmune patients.^27^

Principally, the degree and duration of the impact of inflammation on the vascular walls is considered inherent to the accelerated atherosclerosis present in RA.^28,29^ However, conventional CVD risk factors, such as smoking, dyslipidaemia, diabetes mellitus (DM) and hypertension also inflate the CVD risk of RA patients, who notably present lower levels of HDL and more frequent DM onset than the general population.^30^ As a consequence, the latest therapeutic guidelines released from the European League Against Rheumatism (EULAR) underline the importance of modifying established CVD risk factors apart from adequately regulating RA-induced inflammation.^20^ While the former can be accomplished through lifestyle changes and targeted therapeutic interventions, the latter often necessitates the use of disease modifying anti-rheumatic drugs (DMARDs), whose burden on the cardiovascular system should also be reckoned with.

### Non-pharmacological interventions

Although commonly underestimated by clinicians, lifestyle modifications have the capacity to significantly lower the overall CVD risk of RA patients, contributing to high-level primary or secondary prevention of cardiovascular events. General recommendations apply regarding maintaining optimal body weight and adopting a healthy diet.^31^ Unfortunately, behavioural changes towards maintaining a healthy lifestyle are not always successful even with appropriate patient education, and need to be consistently advocated by the treating physicians.^32^

A major behavioural intervention that has been manifold proven effective in abating CVD risk in RA is exercise. Its potent beneficial effects are partially owed to its anti-inflammatory properties, as it has been reported to affect markers of disease progression and enhance the functional status of RA patients.^33^ High intensity aerobic exercise has been correlated with a significantly decrease in blood pressure, body mass index and disease severity,^34^ yet even low intensity training has the capacity to modify CVD risk. Moderate-intensity physical activity (ie, behaviour ≥ 3 metabolic equivalents daily) is recommended for patients with RA. This can alleviate inflammatory disease activity, decrease joint pain and fatigue and improve physical function.^35^ Unfortunately, RA patients often maintain sedentary lifestyle.^35^ Apart from the well-documented generic effects of physical activity on disease parameters, exercise programs tailored to the needs of each patient have the potential to improve cardiorespiratory and endothelial functions at an individual level, exerting favourable effects on the vasculature. Additionally, the psychological benefits of exercise should not be overlooked. As a consequence, Centres for Disease Control and Prevention (CDC) recommendations for RA patients include four physical activity programs.^36^ Nevertheless, the multifaceted effects of exercise interventions in patients with RA remain consistently underreported in the existing literature. To this end, the IMPACT-RMD toolkit has been recently proposed for the design and reporting phase of every trial reporting on exercise dosage in patients with rheumatic and musculoskeletal diseases.^37^

The use of tobacco is a major modifiable CVD risk factor in RA. Apart from its burden on the cardiopulmonary system, smoking constitutes a significant risk factor for RA onset and progression, as it is known to increase the disease burden in RA and is held responsible for increased disease severity and poor clinical outcomes.^38^ This comes down to the fact that smoking upregulates the generation of anticitrullinated antibodies, thus causing rheumatoid factor positivity and compromising patients’ response to treatment, thereby increasing the overall CVD morbidity.^39,40^ According to a meta-analysis of longitudinal data collected from RA patients, the frequency of CV events was increased by 50% in the smoking compared to the non-smoking cohort.^41^

### Cardiovascular aspects of RA therapies

Antirheumatic drugs comprise a wide spectrum of agents including DMARDs, steroids and nonsteroidal anti-inflammatory drugs. Despite their substantial contribution on the elimination of inflammatory aspects involved in the CVD risk in RA, antirheumatic drugs may often exert adverse effects on the cardiovascular system, such as increases in blood pressure or lipid levels.^42,43^ These undesirable actions should also be taken into consideration and co-calculated with the person-alised CVD risk of each patient when deciding on an optimal therapeutic regimen.

Disease control has been tightly correlated with fewer CV events, since a decline of the clinical disease activity index (CDAI) by 10 points has been proven to reduce CVD risk in RA by 20%.^25^ Although optimal disease control seems to sufficiently reduce CVD risk regardless of the antirheumatic agent used, some types of therapy seem to be more beneficial than others.

For instance, methotrexate has been reported to reinforce the anti-inflammatory properties of HDL cholesterol, decreasing the overall CVD risk of RA patients by about 20% according to a meta-analysis.^44,45^ Methotrexate may modify CVD risk and provide a substantial survival benefit, largely by reducing cardiovascular mortality, compared to other conventional DMARDs.^46^ Antimalarial drugs such as hydroxychloroquine have also been found to exert favourable effects on the cardiovascular system by downregulating inflammatory pathways, leading to a decrease of CVD events exceeding 70%, although this evidence has not been unanimous as conflicting findings were reported by another trial.^47^

On the other hand, glucocorticoid exposure has been correlated with increased CVD risk, both in a dose and time-dependent manner. An increase of glucocorticoid use of 5mg daily has been found to inflate CVD risk by 13% while also being linked to an overall increase of cerebrovascular events and type 2 DM onset, as well as reduced survival rate.^48^ It is worth noting that both the instant effect and the cumulative burden of steroids have been associated with increased risk of myocardial infarction.^49^ Consequently, EULAR guidelines focusing on CVD risk management have accommodated the concept of avoiding or tapering steroid use to the minimal use the soonest possible. Accumulating evidence on the impact of RA treatments on the CVD risk of patients has shed light onto the deleterious effects of cyclooxygenase (COX) inhibitors and NSAIDs on patients’ vasculature. NSAIDs can be divided into two large categories, namely, non-selective and selective COX inhibitors, that are commonly used to achieve pain relief and inflammation control. Nonetheless, these drugs have been linked with burdensome effects on the cardiovascular system, increasing the risk for congestive heart failure, myocardial infarction, and sudden death.^50^ Furthermore, these drugs often lead to the emergence or exacerbation of hypertension, a traditional aggravating factor of CVD risk.^51^ Evidence on the relative risk of each of these agents remains conflicting, as in the case of naproxen, a non-selective COX inhibitor.^52^ Although the case of rofecoxib was one of the most studied for its harmful properties, the CVD burden caused by celecoxib use was found to be similar to that of ibuprofen or naproxen.^53^ Thus, the use of such drugs is to be limited at lowest possible frequency and dosing.

Biological DMARDs may offer a CVD risk benefit, especially those mediating the downregulation of TNFa pathways.^47^ At the same time, DMARDs including biologics are also known to increase lipid levels.^54^ It remains unclear whether biological DMARDs exert direct effects on the cardiovascular system, or whether any favourable effects are in fact mediated by adequate control of systemic inflammation.^55^ Further research is needed on the cardiovascular aspects of novel biological DMARDs, specifically Janus kinase (JAK) inhibitors. Administration of JAK inhibitors in patients with RA was associated with elevated risk of major adverse CVD events, cancers and several adverse events compared to a TNF inhibitor,^56^ although the exact mechanisms of action on the cardiovascular system remain under investigation.^57^

### Control of comorbidities

Appropriate management of traditional CVD risk factors emerges as extremely important in patients with RA. Hypertension and DM have been associated with a twofold rise of CVD risk, while hypercholesterolemia can cause CVD morbidity to escalate by 73%.^58^ It is worth noting that the prevalence of established CVD risk factors is high among RA patients, pointing towards a shared pathogenesis and rendering their proper management even more critical.^59,60^ Once identified, these factors should be closely monitored and efficiently controlled, initially with lifestyle interventions along with medication regimens as indicated, to achieve treatment targets for blood pressure, glycated haemoglobin, and lipids. Of note, alterations in disease activity and antirheumatic therapy should be taken into account before the initiation or titration of such regimens, with awareness that immunosuppressive therapy may significantly impact established CVD risk factors in some patients.^47^ Deplorably, these factors are reported to be sub-optimally treated in more than half of the RA population.

### Hypertension

European Society of Hypertension guidelines define hypertension as persistent systolic blood pressure and/or diastolic blood pressure elevation above 140/90 mmHg, while US guidelines are stricter considering individuals with systolic and/or diastolic blood pressure above 130/80 mmHg as hypertensive.^61^ Accumulating evidence suggests that the prevalence of hypertension is increased among RA patients, compared to the general population, reaching 70%. However, only 25% of RA subjects receiving antihypertensive manage to reach the recommended treatment targets.^62^ Regarding blood pressure control, treatment goals for RA patients do not differ from those recommended by international hypertension guidelines for the general population. These can be summarised as follows; for hypertensive patients <65 years old with comorbidities blood pressure thresholds of less than 130/80 mmHg are recommended, whereas for patients > 65 years blood pressure should be lower than 140/80 if tolerated. By contrast, US guidelines recommend reduction of blood pressure below 130/80 for all individuals.^63^

According to EULAR suggestions, blood pressure management in RA should comply with the national CVD preventive recommendations for the general population without necessarily including a certain class of agents.^20^ A variety of antihypertensive drugs could be eligible for this use, while the combination of two agents is advisable. Preferably, a drug acting on the renin-angiotensin-aldosterone system such as angiotensin converting enzyme (ACE) inhibitors and angiotensin II receptor blockers (ARBs) are drugs of choice for hypertensive RA patients with high plasma renin activity occurring due to increased sympathetic tone. This class of drugs offers additional benefits, downregulating proinflammatory markers, and boosting the expression of anti-inflammatory mediators.^64,65^ Monotherapy is usually adequate in low-risk subjects with SBP<150 and for frail elderly patients. In the opposite case, RAS inhibitors can be co-administered with a calcium channel blocker or a diuretic to achieve a synergistic effect. The inclusion of beta-blockers applies to patients with a history of angina, myocardial infarction, heart failure or arrhythmias. Beta-blockers along with calcium channel blockers are also suitable for women with child-bearing potential.^63^ To address Raynaud’s phenomenon, the use of calcium channel blockers or RAS inhibitors is advisable, while the vasodilatory effect of non-selective beta blockers such as carvedilol and nebivolol could also be employed. Resistant hypertension could be treated with the addition of a third agent to the antihypertensive regimen. Regardless of the antihypertensive medication administered, its effects can be assessed within two months after treatment initiation and modified in the case of electrolyte imbalances or kidney dysfunction.^4^

### Type 2 diabetes mellitus

The incidence of metabolic syndrome, prediabetes, and DM has been reported to be particularly increased in patients with inflammatory mediated immune disorders including RA.^66^ The high prevalence of insulin resistance in RA has been attributed to the concurrence of active inflammation along with exposure to glucocorticoids and adiposity.^67^ In fact, an increase of glucocorticoid use by 5mg has been associated with 30% increase of type 2 DM onset, while a daily administration of over 7.5 mg has been shown to exert detrimental effects on glucose metabolism.^68^ However, there is a lack of large-scale studies comparatively assessing the effectiveness of various glucose-lowering drugs in RA. As in hypertension, glycaemic control in RA should follow current guidelines for the general population to accomplish a HbA1c target of <7% that has been linked with long-term prevention of hypoglycaemia and vascular complications of DM.

### Lipids and lipoproteins

Dyslipidaemia often complicates the course of accelerated atherosclerosis in RA and needs to be taken into account in CVD risk management.^69^ Still, many patients with RA with dyslipidaemia remain undertreated.^70^ Remarkably, dyslipidaemia follows a different pattern under the impact of chronic inflammation in the course of RA, also known as the “lipid paradox”. More specifically, RA patients with active disease present low levels of TC, LDL-C, and HDL-C, which increase with antirheumatic therapies that effectively suppress inflammation. However, both higher and lower lipid levels in RA have been paradoxically associated with increased CVD risk compared to moderate levels in a U-shaped relationship. Therefore, it is reasonable to assess lipid profile in patients with RA when disease activity is stable or in remission.^71^

The use of statins has been found to exert positive effects in RA patients, contributing to arthritis control, modulating their lipid profile and boosting RA drug effectiveness. Statin cessation was further linked to increased CVD mortality due to the absence of their lipid-lowering and anti-inflammatory effects.^72^ This class of agents has been shown to revert arterial stiffness.^73^ Statin initiation should however depend on the risk profile of each patient, taking into account drug interactions, adverse events and prospective benefits rather than solely adhering to LDL target values.^74^ Talking of lipids, several RA therapies such as tocilizumab and tofacitinib require lipid profile assessment after their initiation. Regarding glucocorticoids, prednisone has been found to increase HDL levels, while leaving the rest lipid parameters unaffected. Lastly, methotrexate was also found to enhance the lipid profile of patients probably owing to better disease control.^75,76^

## CONCLUSIONS

CVD often complicates the course of RA, exacerbates physical disability, and limits life expectancy. It is imperative to identify patients at increased risk for CVD. Unfortunately, yet neither population-based nor RA-specific CVD risk calculators have accomplished successful CVD risk prediction when tested in real-life cohorts. While CVD risk calculation in rheumatologic patients remains problematic, frequent screening for CVD risk factors and comorbidities appears reasonable towards their early identification and subsequent management. A meticulous evaluation of CVD risk factors along with the disease burden (e.g., disease flares and remissions, disease duration and activity) is hence warranted on a patient-by-patient basis. Management of CVD risk in RA comprises two major pillars: adequate disease control and appropriate management of CVD risk factors and comorbidities. The cardiovascular effects of antirheumatic medications need to be further taken into account. There is notwithstanding lack of a consortium that brings together different specialties engaged in the care of patients suffering from RA (e.g., rheumatologists, cardiologists, general practitioners, etc). Thus, general recommendations from relevant scientific societies (cardiology, lipidology, hypertension, diabetology) apply for treatment strategies and treatment goals, taking into consideration the individual patient characteristics as mentioned above.
